# Preparation and Electrochemical Characterization of Mesoporous Polyaniline-Silica Nanocomposites as an Electrode Material for Pseudocapacitors

**DOI:** 10.3390/ma8041369

**Published:** 2015-03-25

**Authors:** Lei Zu, Xiuguo Cui, Yanhua Jiang, Zhongkai Hu, Huiqin Lian, Yang Liu, Yushun Jin, Yan Li, Xiaodong Wang

**Affiliations:** 1 Beijing Key Laboratory of Specialty Elastomer Composite Materials, College of Materials Science & Engineering, Beijing Institute of Petrochemical Technology, Beijing 102617, China; E-Mails: zulei@bipt.edu.cn (L.Z.); jiangyanhua@bipt.edu.cn (Y.J.); huzhongkai@bipt.edu.cn (Z.H.); lianhuiqin@bipt.edu.cn (H.L.); yang.liu@bipt.edu.cn (Y.L.); jinyushun@bipt.edu.cn (Y.J.); liyan@bipt.edu.cn (Y.L.); 2 State Key Laboratory of Organic-Inorganic Composites, Beijing University of Chemical Technology, Beijing 100029, China

**Keywords:** polyaniline, mesoporous silica, nanocomposites, vapor phase approach, specific capacitance

## Abstract

Mesoporous polyaniline-silica nanocomposites with a full interpenetrating structure for pseudocapacitors were synthesized via the vapor phase approach. The morphology and structure of the nanocomposites were deeply investigated by scanning electron microscopy, infrared spectroscopy, X-ray diffraction, thermal gravimetric analysis and nitrogen adsorption-desorption tests. The results present that the mesoporous nanocomposites possess a uniform particle morphology and full interpenetrating structure, leading to a continuous conductive polyaniline network with a large specific surface area. The electrochemical performances of the nanocomposites were tested in a mixed solution of sulfuric acid and potassium iodide. With the merits of a large specific surface area and suitable pore size distribution, the nanocomposite showed a large specific capacitance (1702.68 farad (F)/g) due to its higher utilization of the active material. This amazing value is almost three-times larger than that of bulk polyaniline when the same mass of active material was used.

## 1. Introduction

As a new kind of electrochemical energy storage device, supercapacitors have attracted great attention, both in academic and practical applications, due to their excellent properties, such as sustainable cycling life, fast charging-discharging rate, high specific power and excellent cycle stability [1,2,3,4,5,6,7]. With these merits, supercapacitors would fill the gap between conventional electrochemical capacitors and batteries. It is well known that depending on the different charge-storage mechanism, supercapacitors could be defined as either electrical double-layer capacitors or pseudocapacitors, in which the pseudocapacitance results from the Faradaic reactions occurring at the very nearly electrode interface and utilizing electroactive materials to store energy through redox reactions [8,9].

The electrode material is an important factor to directly decide the capability, delivery rates and efficiency of the pseudocapacitors [10]. Various organic or inorganic materials could act as the pseudocapacitor electrode materials, including carbonaceous materials [5,11,12], transition-metal oxides [13] and conductive polymers [14,15,16]. Among the different conductive polymers, polyaniline (PANI) is believed to be the ideal candidate for supercapacitors due to its good redox reversibility, electrical conductivity, easy synthesis procedure and good environmental stability [17,18,19,20].

In recent years, some reports have presented that a material in the nano-size form with a large surface area and high porosity gives an excellent performance as the electrode material for supercapacitors [12,21,22,23,24]. According to this, great efforts have been devoted to developing high-performance nanocomposites, which were composed of PANI and other organic or inorganic materials [25,26,27,28,29,30,31,32,33,34,35,36,37,38,39]. These novel nanocomposites commonly have a relatively large specific surface area and appropriate pore diameter distribution, which could offer the possibility to improve the specific capacitance of supercapacitors due to their larger exposure area and more active Faradaic reactions sites.

As a classical inorganic material, mesoporous silica has wide applications due to its high specific surface area, large pores, tunable porosity and high chemical stability [40,41,42,43]. Therefore, a composite electrode formed by mesoporous silica and PANI may possesses a good electrochemical activity and relatively large specific surface area to improve the performance of supercapacitors. However, for a PANI-silica nanocomposite, if the PANI chains were isolated in the channels or pores of mesoporous silica and could not form a conductive network, the electrical performance of the composite would be decreased very greatly, due to the free movement of electrons being restricted within the PANI chains [44]. Therefore, a nanocomposite of PANI and mesoporous silica preferably has a consecutive organic conductive network with a relative large effective specific surface area, if it were used as the pseudocapacitor electrode material.

Apart from the electrode materials, the electrolyte is also particularly important in affecting the performance of supercapacitors. Some papers published in recent years have proposed that the total capacitance and energy density of supercapacitors would be improved greatly if an electrochemically-active material were added into the electrolyte. These electrochemically-active materials include indigo carmine [45], hydroquinone [46], methylene blue [47], *etc.* The excellent electrochemical performance of supercapacitors could be ascribed to an additional pseudocapacitive contribution of the electrochemically-active materials. According to these results, an excellent electrochemical performance of supercapacitors would be obtained by utilizing a large effective specific surface area nanocomposite electrode material that possesses a consecutive conductive network, and a new composite electrolyte should also preferably be used.

Herein, we synthesized a novel mesoporous PANI-silica nanocomposite electrode material through the vapor phase approach. The mesoporous nanocomposites possess a full organic-inorganic interpenetrating structure, leading to a consecutive PANI conductive network, and the specific surface area is 81.08 m^2^/g, which is much larger than that of the bulk PANI. The largest specific capacitance was 1702.68 F/g when potassium iodide was added into the electrolyte. This excellent value was almost three-times larger than that of the bulk PANI when the same weight of active material was used.

## 2. Results and Discussion

**Figure 1:**
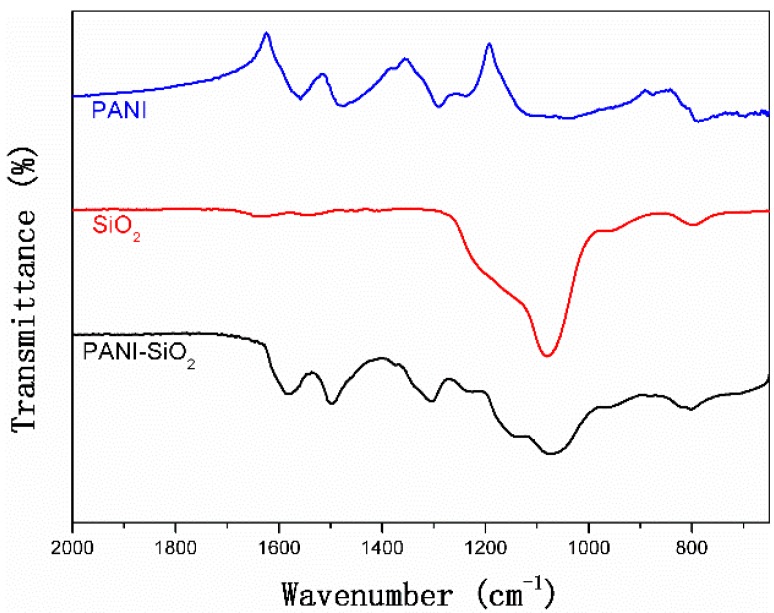
FTIR spectra of bulk polyaniline (PANI), mesoporous silica and PANI-silica.

Figure 1 presents the FTIR spectra of bulk PANI, mesoporous silica and PANI-silica nanocomposite. Similar to the mesoporous silica, the PANI-silica shows a typical Si-O-Si antisymmetric and symmetric stretching at 1078 cm^−1^ and 801 cm^−1^, respectively, indicating the existence of silica. Except for the original peaks involved with silica, some new absorption peaks could also be observed in PANI-silica. The peak at 1305 cm^−1^ could be assigned to the C-N stretching vibration of the benzenoid ring, and the peak at 1157 cm^−1^ is attributed to the aromatic C–H in-plane bending. The peaks centered at 1498 cm^−1^ and 1589 cm^−1^ are due to the C = C and C = N stretching of the benzenoid and quinoid rings, respectively. These typical absorption peaks confirm the existence of PANI in the nanocomposite. However, when comparing the spectra of the bulk PANI and PANI-silica, the typical adsorption peaks of PANI all blue-shift to higher wavenumbers, as shown in Figure 1. This phenomena implies that the PANI in PANI-silica may be restricted by the silica; in other words, the PANI probably penetrates into the mesoporous silica pores and channels [48].

**Figure 2:**
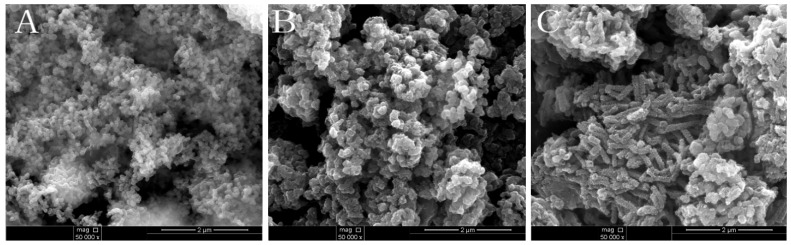
SEM images of samples: (**A**) mesoporous silica; (**B**) PANI-silica; and (**C**) bulk PANI.

The SEM images of mesoporous silica, bulk PANI and PANI-silica are presented in Figure 2. As can be seen in Figure 2A, the mesoporous silica has a spherical particle morphology, and the surface is smooth. However, as shown in Figure 2B, the PANI-silica has a rough irregular surface, indicating that polymerization perhaps occurred both in the internal channels and pores and external surface of the mesoporous silica. The bulk PANI shows a rough short stick morphology, as presented in Figure 2C.

**Figure 3:**
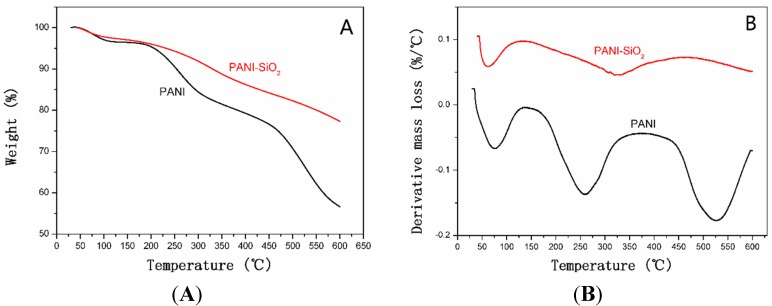
(**A**) TGA curves of bulk PANI and PANI-SiO_2_. (**B**) DTG curves of bulk PANI and PANI-silica.

TGA and DTG curves of bulk PANI and PANI-silica nanocomposite are presented in Figure 3. As shown in Figure 3A, the bulk PANI demonstrates a characteristic three-step weight loss process, and about 55% of PANI was left due to the carbonization under nitrogen atmosphere. The initial loss is owed to the elimination of absorbed water and the weight loss between 150 °C and 300 °C is attributed to the decomposition of dopant molecules. The last weight loss process corresponds to the degradation of PANI. Furthermore, it also can be found that the total mass loss of PANI-silica is about 23%, including the elimination of water and the decomposition of PANI. Correspondingly, the content of silica is almost 77% in PANI-SiO_2_. However, as shown in Figure 3B, the PANI-silica possesses a typical two-step decomposition. At a temperature below 100 °C, the elimination of water occurs, and the weight loss is about 3%. The following weight loss process is apparently due to the decomposition of PANI, and the weight loss is nearly 20%. It is notable that the residues of bulk PANI and mesoporous PANI-SiO_2_ are not stable, even when the temperature was increased to 600 °C, this result could be ascribed to the high crystallinity of bulk PANI and the existence of silica [49]. Compared with the bulk PANI, the polymer in the PANI-silica possesses worse thermal stability, which may be due to the PANI in the nanocomposite being amorphous and the silica having a restriction on the PANI [48].

**Figure 4:**
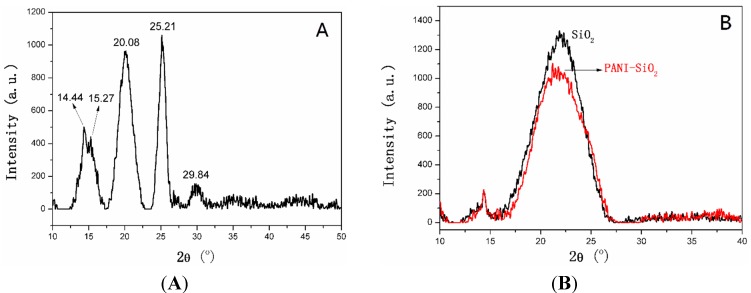
(**A**) X-ray scattering patterns of the bulk PANI. (**B**) X-ray scattering patterns of mesoporous silica and PANI-silica.

The X-ray scattering patterns of the bulk PANI, mesoporous silica and PANI-silica are exhibited in Figure 4. As demonstrated in Figure 4A, the main diffraction peaks of the bulk PANI at 2θ = 14.4°, 15.27°, 20.1°, 25.2° and 29.8°, respectively. The diffraction peaks at 15.27°, 20.1°, 25.2° correspond to the (011), (020) and (200) crystal planes of orthorhombic crystalline PANI in its emeraldine salt form, respectively [50]. The peak at 20.1° is related to the repeat units of the polyemeraldine chain and the periodicity parallel to the polymer chains of PANI. The peak at 25.2° is owed to the periodicity in the direction perpendicular to the polymer chain [51]. These typical diffraction peaks confirm that the PANI is highly crystalline. The wide angle X-ray diffraction (WAXD) patterns of mesoporous silica and PANI-silica are presented in Figure 4B. It can be seen clearly that the silica possesses a broad diffraction peak at about 21.9°, indicating that the mesoporous silica is amorphous. Similarly, the diffraction peak of PANI-silica did not have an apparent shift, but only the diffraction intensity is lower than mesoporous silica. This result may be attributed to relatively low scattering contrast between the pores and walls of mesoporous silica resulting from the formation of PANI chains in the channels of silica [52]. Furthermore, there is no diffraction peak of PANI emerging in the patterns of PANI-silica, confirming that the PANI is also amorphous and has been encapsulated in the pores and channels of the silica; and the crystallization of PANI is impeded owing to the confinement of silica framework.

**Figure 5:**
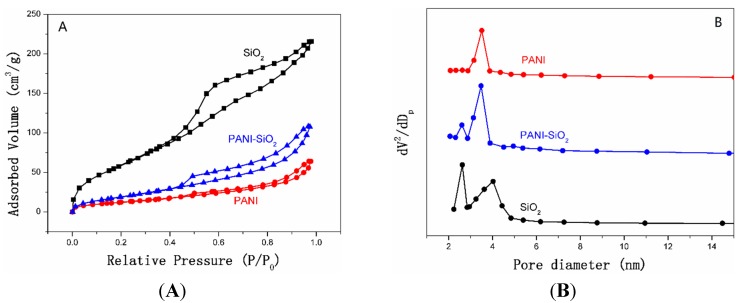
(**A**) N_2_ adsorption/desorption isotherms of mesoporous silica, PANI-silica and bulk PANI. (**B**) Pore size distribution curves of mesoporous silica, PANI-silica and bulk PANI.

The N_2_ adsorption/desorption isotherms and pore size distribution curves of mesoporous silica, PANI-silica nanocomposite and bulk PANI are shown in Figure 5, and detailed data are listed in Table 1. From Figure 5A, it can be seen clearly that the silica has a typical Type IV isotherm and displays a distinct hysteresis loop of H2 in the range of 0.4–1.0 P/P_o_ (P is the partial pressure of the adsorbate and the P_o_ is adsorbent saturated vapor pressure). The pore size distribution curves are exhibited in Figure 5B. As can be seen clearly, the pore diameter distribution curve of the silica calculated from adsorption branches shows two sharp peaks at 2.6 nm and 4.0 nm, respectively, suggesting that the silica possesses two pore structures. A similar result could also be found in PANI-silica, the peaks centered at 2.6 nm and 3.5 nm. These results prove that the silica and PANI-silica are all mesoporous. However, compared with the mesoporous silica, the PANI-silica has a small capillary condensation step, which appears in the range of 0.8–1.0 P/P_o_, illustrating that the PANI-silica may possess a hollow core or cavity caused by the packing of particles, and this result is also demonstrated by the two different pore size distribution peaks in Figure 5B. As listed in Table 1, the mean pore diameter of PANI-silica and mesoporous silica is 4.37 nm and 6.68 nm, respectively. The smaller mean pore diameter of PANI-silica may be due to the existence of PANI, which exists both in internal pores or channels and the surface of the silica. Although the bulk PANI has a similar isotherm with the PANI-silica, the specific surface area and mean pore diameter are all much smaller than that of the PANI-silica.

According to all of the test results obtained above, it is reasonable to presume that the PANI had penetrated into the silica pores and channels; in other words, the PANI-silica possesses a full interpenetrating structure.

**Table 1:** N_2_ adsorption/desorption measurement results of bulk PANI, mesoporous silica and PANI-silica.

Electrode	S_BET_ (m^2^/g)	D (nm)	V_t_ (cm^3^/g)
SiO_2_	240.33	4.39	0.41
PANI-SiO_2_	81.08	5.72	0.20
PANI	48.11	6.68	0.11

**Figure 6:**
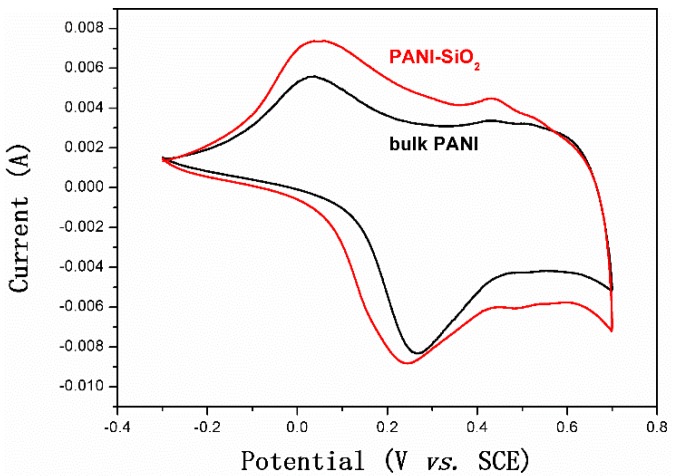
Cyclic voltammetry (CV) measurement results of bulk PANI and PANI-SiO_2_. SCE, saturated calomel electrode.

Cyclic voltammetry (CV) measurements were performed to investigate the electrochemical performance of bulk PANI and PANI-silica nanocomposite in the voltage range from −0.3 to 0.7 V (*vs.* the saturated calomel electrode (SCE)), taken at a scan rate of 5 mV/s. As indicated in Figure 6, the bulk PANI shows a pair of broad and symmetric redox peaks (centered at −0.15 V and 0.33 V), suggesting a typical reversible redox reaction of I^−^/I_3_^−^, which is given by Equation (1) [53]:

3I^−^ − 2e^−^ ↔ I_3_^−^ (1)

A similar behavior is also shown in PANI-silica, the redox peaks centered at 0.15 eV and 0.33e V, respectively. It is well known that the redox reaction rate in electrochemical systems are negatively correlated with the peak-potential separation (Epp), the difference between the anodic peak potential (Epa) and the cathodic peak potential (Epc). The Epp of mesoporous silica and PANI-silica are 0.48 eV and 0.18 eV, respectively, demonstrating that the PANI-silica has a greater electrochemical activity than that of the bulk PANI. This result may be attributed to the PANI-silica possessing a higher doping level than that of the bulk PANI [53]. As listed in Table 1, the PANI-silica has a larger specific surface area and mean pore diameter than bulk PANI, leading to much more electrochemically-active sites [54]. Therefore, the PANI-silica has better electrochemical performance than bulk PANI.

**Figure 7:**
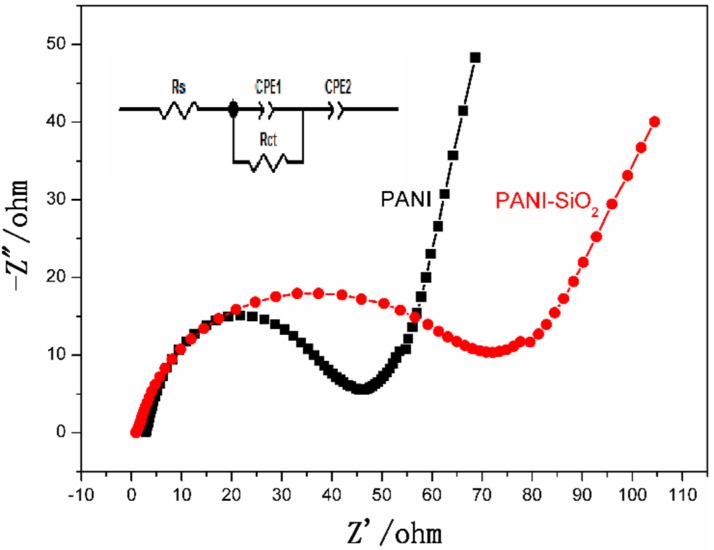
Nyquist plots and equivalent circuit of bulk PANI and PANI-SiO_2_. Rs, serial resistance; CPE, constant phase element.

Electrochemical impedance spectroscopy (EIS) analysis was performed to evaluate the charge transfer character of bulk PANI and PANI-silica nanocomposite. The Nyquist plots and equivalent circuit are presented in Figure 7. In the Nyquist plots, the depressed semi-circle in the high frequency is owed to the electrode possibly blocking the ionic exchange of the Faradic process, which is generated at the electrode/electrolyte interface. The straight line in the low frequency is due to the Warburg diffusion impedance. The diameter of the semicircle presents the difficulty of the charge transfer in the electrochemical system. As exhibited in Figure 7, the impedance curves of bulk PANI and PANI-silica show a single semicircle in the high frequency region and a straight line in the low frequency region. Although the bulk PANI and PANI-silica have a similar impedance spectra, the diameter of the two semicircles is very different. The semi-circle of the PANI-silica is much larger than that of the bulk PANI, suggesting that the PANI-silica has a higher electrochemical charge transfer resistance. In other words, the charge transfer in the PANI-silica/electrolyte is much slower than that of the bulk PANI. This result could be ascribed to the amorphous structure of PANI and the existence of silica in the nanocomposite. It is well known that silica is an inorganic semiconductor, which has a bulking property of ionic and electron transfer. Consequently, the silica may hinder the ionic transportation at the surface of the PANI-silica/electrolyte. Furthermore, the amorphous PANI has poor conductivity compared to the crystallized bulk PANI, leading to a larger internal resistance than that of the bulk PANI [55]. As a consequence of these disadvantages, the PANI-silica has a larger charge transfer resistance than that of bulk PANI. 

The equivalent circuit of the impedance spectra is shown in Figure 7. As can be seen obviously, the equivalent circuit consists of an ohmic serial resistance (Rs) in series with a parallel combination of charge transfer resistance (Rct) and a constant phase element (CPE1), in addition to a series with another constant phase element (CPE2). 

The shape of the Nyquist plots can be considered as an indication of the porous structure behavior, in good agreement with the SEM characterization [56].

Galvanostatic charge/discharge tests were performed to assess the electrochemical capacitance of bulk PANI and PANI-silica. The specific capacitance (Cs) can be calculated according the following Equation (2) [40]:

Cs = (IΔt)/(ΔVm)
(2)

where Cs is the specific capacitance (F/g), I is the current loaded (A), Δt is the discharge time (s), ΔV is the potential window during the discharge process and m is the mass of active material in a single electrode (g).

**Figure 8:**
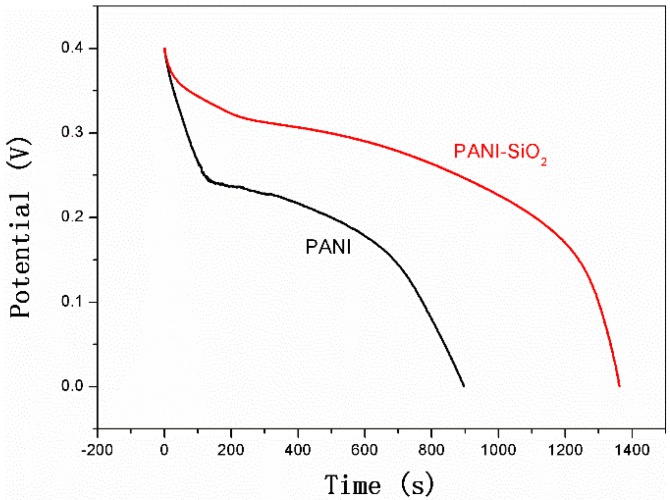
Galvanostatic charge/discharge test results of bulk PANI and PANI-SiO_2_.

As described clearly in Figure 8, the PANI-silica has a longer discharge time than that of the bulk PANI. The discharge time of PANI-silica and bulk PANI is 1,362.2 s and 894.6 s, respectively. According to Equation (2), a Cs value of 1,702.75 F/g is obtained for PANI-silica and 1,118.25 F/g for bulk PANI. Furthermore, with the consideration of only the PANI contributing the electrochemical activity in PANI-silica, the Cs value of PANI-silica is almost three-times larger than that of bulk PANI if the same mass of electroactive material were used. This excellent Cs value of PANI-silica could be owed to the high specific surface and suitable pore diameter of PANI-silica, which led to the higher utilization of the active material.

## 3. Experimental Section 

### 3.1. Chemicals

Oleic acid (OA), 3-aminopropyl-triethoxysilane (APTES), tetraethyl orthosilicate (TEOS), aniline, ammonium persulfate (APS), hydrochloric acid (HCl), acetylene black, polytetrafluoroethylene (PTFE), N-methyl-2-pyrrolidone (NMP) and potassium iodide (KI) were purchased from Aladdin. All of the reagents were used as received, except aniline. The aniline was purified by reduced pressure distillation before it was used.

### 3.2. Sample Preparation

#### 3.2.1. Preparation of PANI

The bulk PANI was prepared by the aqueous polymerization method. In a typical procedure, 0.5 g APS was dissolved in 100 mL of 0.2 M HCl solution and stirred at 0 °C for 30 min. Then, 0.2 g aniline were added to the above solution and stirred for another 2 h, followed by placing at 0 °C for 24 h. The precipitate was filtered and washed with ethanol and water until the filtrate was neutral. Then, the precipitate was dried at 60 °C under vacuum for 24 h. 

#### 3.2.2. Preparation of Mesoporous Silica

The mesoporous silica was synthesized according to [57]. In a typical procedure, 0.564 g OA, 57.65 g deionized water and 4.6 g ethanol were mixed together at room temperature under vigorous stirring. After stirring for 30 min, 2.8 g TEOS and 0.442 g APTES were added to the solution and stirred for another 20 min. Then, the reaction solution was stirred at room temperature for 2 h followed by aging at 80 °C for 24 h. The mesoporous silica was obtained by filtering and washed with ethanol several times in order to remove the OA from the precipitate. Then, the precipitate was dried at 80 °C for 12 h and calcined at 550 °C for 6 h.

#### 3.2.3. Preparation of Mesoporous PANI-Silica Nanocomposites

The mesoporous PANI-silica nanocomposite was prepared through the vapor phase method. In a typical procedure, the mesoporous silica (outgassed at 100 °C for 24 h in advance) was contacted with aniline gas at 40 °C for 48 h and then immersed in the solution of 150 mL HCl (0.2 mol/L) and 1.22 g APS. The reaction solution was vigorously stirred at 0 °C for 2 h and then placed for another 24 h. The obtained nanocomposite was filtered and washed with ethanol and aqueous HCl several times and then dried at 60 °C under vacuum for 24 h.

### 3.3. Characterization

#### 3.3.1. Instruments

Scanning electron microscopy (SEM) observations were performed on a COXEM EM-20 microscope operating at 20 kV. Infrared spectrometry (IR) analyses were performed on a Thermal Nicolet infrared spectrometer. Thermogravimetric analyses (TGA) were carried out using a TA instruments TGA2050 from room temperature to 600 °C, with a heating rate of 10 °C/min under nitrogen. Nitrogen adsorption-desorption isotherms were measured at 77 K using a JW-BK static physisorption analyzer after the samples were outgassed for 24 h at 110 °C. The BET surface area was calculated from the desorption branches in the relative pressure range of 0.05–0.35, and the total pore volume and average pore diameter were evaluated at a relative pressure of about 0.99. Wide angle X-ray diffraction (WAXD) patterns were obtained with a Bruker D8 diffractometer in reflection mode using Cu Kα = 0.154 nm with a voltage of 40 kV. The electrochemical performance was investigated by a CHI 660D electrochemical work station.

#### 3.3.2. Electrochemical Test Method

The electrochemical performance of mesoporous PANI-silica nanocomposite and bulk PANI was studied in a three-electrode test cell. Briefly, the as-prepared material, acetylene black and PTFE were mixed in a mass ratio of 80:10:10 and dispersed in NMP to produce a homogeneous paste. Then, the resulting mixture was coated onto the stainless steel grid (about 1 cm^2^) to fabricate the working electrode. Platinum foil (1 cm × 1 cm) was used as the counter electrode, and a saturated calomel electrode (SCE) was utilized as the reference electrode. The electrochemical measurements were performed in 250 mL mixed electrolyte, which was composed of 1 M H_2_SO_4_ and 0.05 M KI at room temperature. The cyclic voltammetry (CV) tests were carried out between −0.3 and 0.7 V (*vs*. SCE) at scan rates of 5 mV/s. Alternating-current impedance (EIS) tests were performed at the open circuit potential between the frequency ranges from 0.01 Hz to 100 kHz with an AC voltage amplitude of 5 mV. The galvanostatic charge/discharge curves were obtained in the potential range of 0–0.4 V (*vs*. SCE) at 0.5 ampere/g (A/g).

## 4. Conclusions

In summary, a novel mesoporous PANI-silica nanocomposite electrode material was synthesized through the vapor phase approach. The PANI-silica nanocomposite possesses a full interpenetrating structure, leading to a continuous PANI conductive network. The nanocomposite shows a uniform particle morphology, and the mean particle size is 200 nm, approximately. The XRD results confirm that the PANI in the nanocomposite is amorphous, while the bulk PANI possesses a good crystalline structure. The specific surface area and mean pore diameter of PANI-silica are 81.1 m^2^/g and 5.7 nm, respectively, which are much larger than that of the bulk PANI. The specific capacitance of PANI-silica is 1702.75 F/g at a discharge rate of 0.5 A/g in a KI electrolyte solution within the potential range from 0 to 0.4 V (*vs.* SCE). This high specific capacitance is owed to its higher utilization of the active materials, due to a large specific surface area and suitable pore size distribution.
